# Design and Evaluation of a Fully Implantable Control Unit for Blood Pumps

**DOI:** 10.1155/2015/257848

**Published:** 2015-10-25

**Authors:** Kristin Unthan, Felix Gräf, Marco Laumen, Thomas Finocchiaro, Christoph Sommer, Hermann Lanmüller, Ulrich Steinseifer

**Affiliations:** ^1^Department of Cardiovascular Engineering, Institute of Applied Medical Engineering, Helmholtz Institute, RWTH Aachen University, 52074 Aachen, Germany; ^2^Center for Medical Physics and Biomedical Engineering, Medical University of Vienna, 1090 Vienna, Austria

## Abstract

As the number of donor hearts is limited while more and more patients suffer from end stage biventricular heart failure, Total Artificial Hearts become a promising alternative to conventional treatment. While pneumatic devices sufficiently supply the patients with blood flow, the patient's quality of life is limited by the percutaneous pressure lines and the size of the external control unit. This paper describes the development of the control unit of the ReinHeart, a fully implantable Total Artificial Heart. General requirements for any implantable control unit are defined from a technical and medical point of view: necessity of a Transcutaneous Energy Transmission, autonomous operation, safety, geometry, and efficiency. Based on the requirements, a prototype is designed; it incorporates a LiFePo_4_ battery pack with charger, a rectifier for transcutaneous energy transmission, the motor's driver electronics, and a microcontroller which monitors and controls all functions. In validation tests, the control unit demonstrated a stable operation on TET and battery supply and a safe switching from one supply to the other. The overall mean efficiency is 14% on TET and 22% on battery supply. The control unit is suitable for chronic animal trials of the ReinHeart.

## 1. Introduction

In some cases of heart failure, a heart transplant is the only therapy left for the patient. While donor hearts remain the gold standard of treatment, the number of patients greatly exceeds the limited number of donor organs. In cases where no allograft is available, Total Artificial Hearts (TAH) can provide an alternative. Pneumatic devices have successfully served as a bridge to transplant for the last 30 years [1]. However, these pneumatic systems require permanent percutaneous drivelines and a noisy compressor [2]. Thus, the improvement of the patient's quality of life becomes an increasingly significant consideration in the development of new devices, as TAH application is extended to the use as destination therapy.

Fully implantable devices renounce the percutaneous drivelines and transmit the energy into the body of the patient via two coils. Two coils of which one is subcutaneously implanted and the other is secured on the patient's skin are inductively coupled to transmit the energy wirelessly. This so-called Transcutaneous Energy Transmission (TET) evades the risk of driveline infection. Implanted backup batteries allow taking off all external gear for a limited time frame and thereby simplify body care and improve mobility. As a consequence, the patient's quality of life is highly improved by these fully implantable devices.

Up to now, only two devices have been fully implanted into patients: the AbioCor TAH and the Lion Heart Left Ventricular Assist Device (VAD) [3, 4]. Unfortunately both are no longer on the market and none of the recently available devices is fully implantable. Lately, the mayor VAD companies resumed TET system developments to enhance the patient's quality of life [1]. Whenever a TET system is used, a control unit which operates the pump also has to be implanted. This paper focuses on the configuration and design of such an implantable control unit. It provides an overview of the ReinHeart TAH, defines special requirements for implantable electronics, and describes the testing methodology for its evaluation. The validation of the prototype in a mock circulation loop is described and hydrodynamic and electric as well as mechanic attributes are presented. The paper closes with a conclusion and a preview of future work.

## 2. Material and Methods

In the following section, the general setup of the ReinHeart TAH, the control units target system, is briefly described. The requirements for adapting an extracorporeal control unit to an implantable control unit are listed and explained. Finally, the test setup for the validation of the prototype is described.

### 2.1. System Overview

The fully implantable Total Artificial Heart ReinHeart is currently being developed at the Institute for Applied Medical Engineering in Aachen, Germany. A detailed description of the ReinHeart can be found in [5]. Unlike a pneumatic drive, the electromagnetic driver of the ReinHeart enables the energy transmission through a TET system. It transmits energy into the body from an external coil to an implanted coil. An alternating current in the external coil induces an electrical voltage in the implantable coil via inductive coupling and supplies the driver with energy with no tissue defects and minimized risk of infection. A paper about the TET system is currently in preparation.

**Figure 1:**
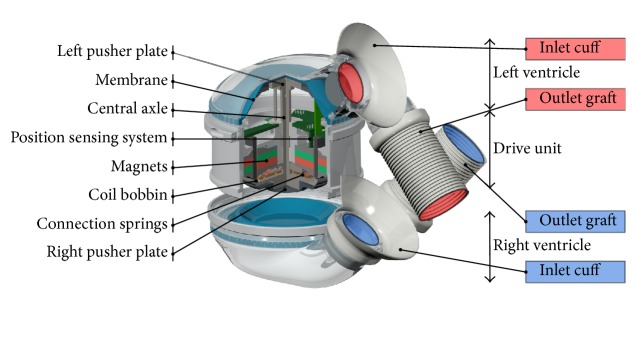
Detailed description of the TAH pump unit [5].

The general setup of the ReinHeart pump unit consists of a left and a right ventricle (Figure 1). The electromagnetic motor is arranged between the ventricles and separated from the blood by a polyurethane membrane. The motor moves two pusher plates which are connected on both sides of an axis. Thus, the pusher plates move simultaneously and alternately eject the left and right chambers. The left systole takes place during the right diastole and vice versa. Since the membranes are not attached to the pusher plates, the ventricles fill passively. The pressure in the compliance chamber is adjustable. Therefore the pressure gradient between the atrium and the motor unit can be increased to control the filling characteristics.

The movable part of the motor consists of the pusher plates on both sides of an axis and a coil bobbin. It is arranged in the magnetic field of the stator. Lorenz force is produced by current flow perpendicular to the magnetic field of the motor magnets and results in an upward motion. Inverting the current in the same magnetic field reverses the force and produces a downward motion. The magnitude of the force can be directly influenced by the amount of current in the coils. Therefore the control unit must provide time dependent current profiles to each coil to maintain the movement of the pusher plates. For a smoother and more efficient movement the motor of the ReinHeart TAH consists of four separate coils. Details about the drive unit are described in [6].

### 2.2. Requirements

When replacing a motor control unit from an external setup with a fully implantable control unit, additional functions must be added and novel requirements have to be taken into account. Those requirements are specified in the following.

#### 2.2.1. Wireless Energy Transmission

All energy used by the pump unit and electronics must be induced into the body by inductive coupling. For energy transmission, a changing flux density is required. As such, alternating current is applied. The alternating current in the external coil induces alternating voltage in the implantable coil, which must be rectified to a stable direct current.

#### 2.2.2. Safety

The driver electronics must be powered at all times after implantation. In the case of a sudden disconnection of the transmitting coil, the power demand of the electronics must be met by an implanted battery pack. In this scenario, the power supply must switch to battery support uninterruptedly. The battery must be charged whenever the TET System is reconnected. The internal battery is expected to supply the TAH for at least half an hour [7]. This will enable the patient to take off all external gear for a period of time which will offer improved quality of life. Finally, the battery has to be safe and durable despite the large number of charging cycles [8]. Consequently, the chemical type of the battery must be chosen accordingly.

#### 2.2.3. Autonomous Pumping

Independent of the connection or disconnection of the external devices, the control unit has to assure a continuous pumping of the TAH, that is, a sinusoidal movement of the pusher plates at a preset beat rate. The movement has to be uniform regardless of varying input and output blood pressures.

#### 2.2.4. Efficiency

The efficiency of the implanted electronics ultimately affects the amount of energy which has to be transmitted into the body. A good efficiency prolongs the time in which the system can support the patient without external components. Furthermore, a low energy consumption and a high efficiency reduce the internally generated heat to a minimum. For comparison, efficiencies reported by earlier developments varied between 6 and 39 % with most efficiencies in the range of 10 to 15% [4, 9, 10].

#### 2.2.5. Geometry

**Figure 2:**
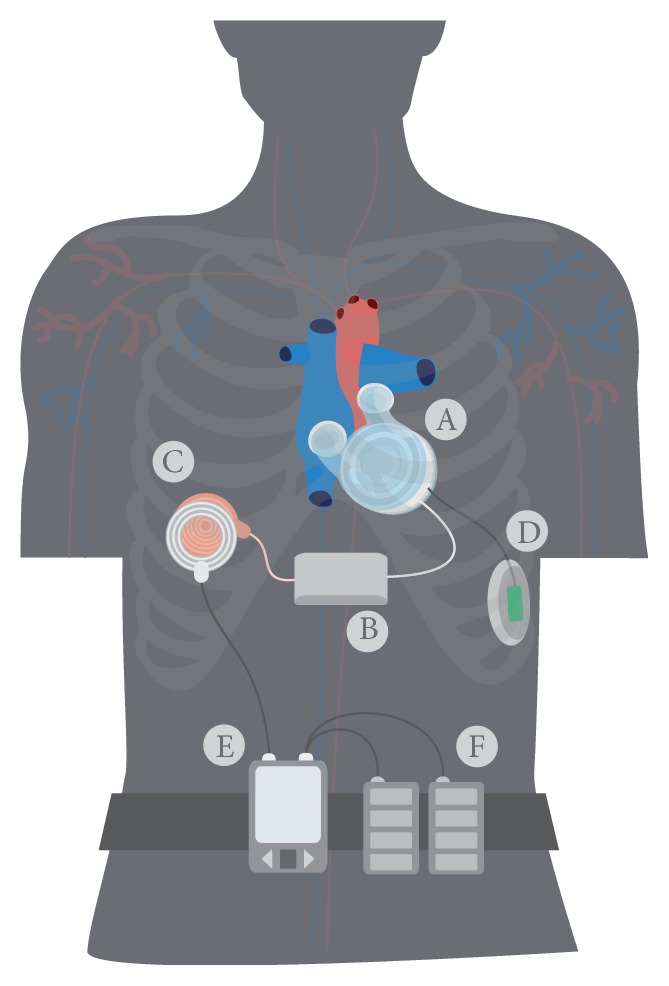
Assembly of the complete TAH system [5].

Anatomical fitting studies, both virtual and cadaver studies, were conducted to determine the optimal size and location of the pump unit in a former study [11]. As a result, the TAH motor unit and ventricles have a diameter of 85 mm and a height of 90 mm. Because the space of the native heart is taken up completely by the pump unit, the electronics must be placed in a separate casing, which is located in the abdomen. To minimize the invasive approach, the electronics and the internal batteries must fit in the same casing. The resulting device configuration is displayed in Figure 2.

The housing volume documented by earlier developments is 277 cm^3^ to 402 cm^3^ [4, 12]. The material of the housing must be biocompatible and seals must protect the electronics from penetration of moisture. Finally, a convex shape, orientated on the shape of the abdominal wall, at the ventral side of the housing and rounded edges makes it more comfortable to wear.

Some of the above mentioned requirements compete against one another, especially at the interplay of efficiency and geometry, which must be considered throughout the whole design. As an example, additional functions like TET or battery support increase the size of the control unit's circuit board. Consequently, the higher number of electrical components, which are included for enhanced functionality, lower the efficiency. In the tradeoff between safety and geometry, not all batteries with a high energy to volume ratio are suitable for implant application. Shape and dimensions of the batteries limit the space available for electronics. Finally, produced waste heat could be spread more effectively by components in bigger packages with a larger surface area.  Table 1 gives an overview of the tasks caused by the requirements. A balance between the different requirements was considered during the design of the control unit, which is described in the following section.

### 2.3. Validation

The designed prototype was validated in a mock circulation loop (MCL) described in detail elsewhere [13]. Systemic and pulmonary resistance, systemic arterial and pulmonary arterial compliance, and the venous volume of the MCL were controlled by a computer. The MCL was filled with a mixture of water (57.5%) and glycerin (42.5%); the resulting viscosity of 3.663 mPas was measured at room temperature. For this evaluation the pressure levels were fixed to the following values. The mean aortic pressure and the pulmonary artery pressure were set to 100 mmHg and 25 mmHg, respectively. The right and left atrium pressures were kept at 10 mmHg mean pressure. All pressures were registered by DPT-6000 (CODAN pvb Critical Care GmbH, Germany) sensors over fluid filled pressure lines. The beat rate was adjusted between 100 and 160 beats per minute. Ultrasound flowmeters (Transonic, USA) captured the resulting flow. All mentioned data was gathered by a dSpace Data Acquisition System (dSpace, Germany). Additional electrical characteristics were recorded by a power meter WT1800 (Yokogawa, Japan) and a keysight oscilloscope (Agilent, USA).

## 3. Results and Discussion

A prototype based on the requirements for an implantable control unit was designed and evaluated. The requirements indicated that additional electronics besides the driver electronics have to be combined to one input power unit. Specifically, the rectifier for the TET, the battery, battery charger, and electronics for switching the input power from battery to TET. A microcontroller takes over the control and monitors functions of the system. Design and evaluation are described separately for each module in the following.

### 3.1. Design of the Prototype

**Figure 3:**
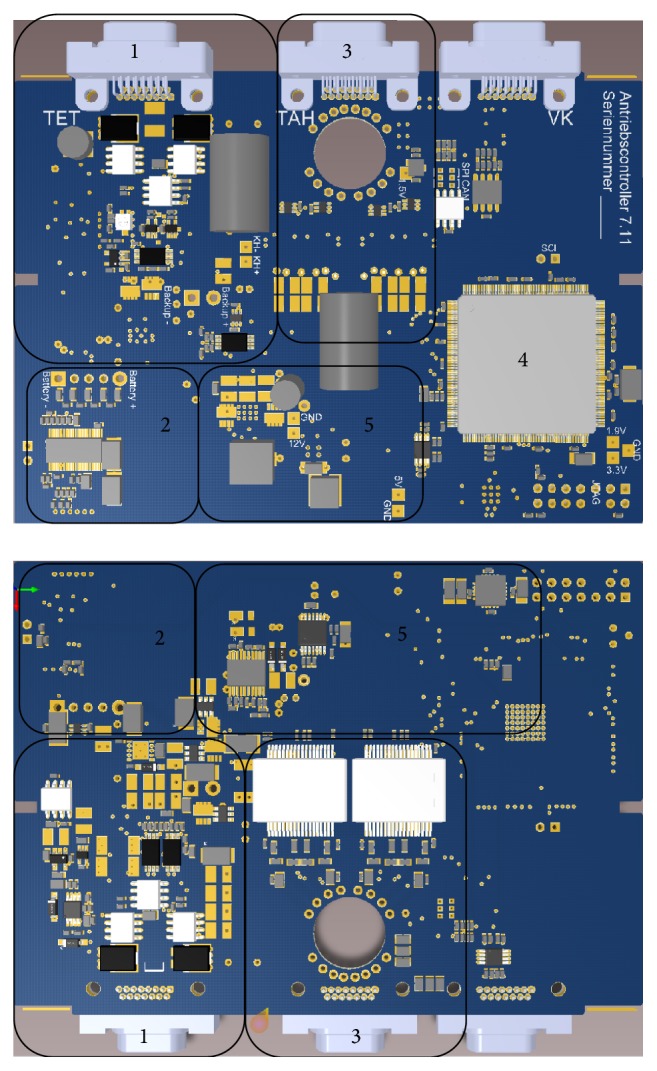
Circuit board of TAH control unit: 1-input power, 2-battery charger, 3-driver electronics, 4-microcontroller, and 5-voltage level shifter.

The design of the electronics was realized with the software Altium Designer. It laid out on a six layer circuit board (Figure 3), because additional layers allow a more compact as well as noise optimized design.  Table 1 summarizes the influence of each initially defined requirement on the different electronic units of the prototype. In the following, the resulting design of each unit is described.

#### 3.1.1. Input Power

The control unit must be continuously powered after implantation.

**Figure 4:**
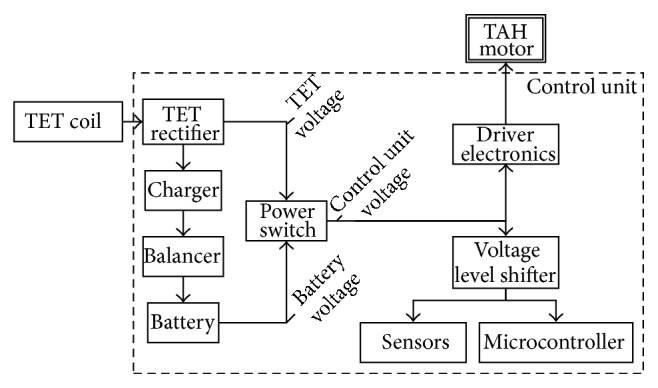
Organization of the input voltage.

Figure 4 documents the organization of the input power. The input switches between an alternating voltage received by the implanted TET coil and the direct voltage of the implanted batteries. Cells consisting of lithium-iron-phosphate (LiFePo_4_) with a capacity of 1.1 Ah were chosen for the internal battery, as this type of a lithium ion battery combines a high power density, low self-discharge rate, a long cycle life, and a safe operation [14]. Since the electronics work with a minimum nominal voltage of 12 V, four cylindrical LiFePo_4_ cells with a single nominal voltage of 3.3 V are used in series. Depending on the state of charge, the battery pack voltage, in the following named battery voltage, reaches 12 to 14.4 V. As this battery voltage can directly supply the power electronics without a voltage level shifter, accompanying power losses were minimized.

The AC voltage of the TET is rectified to a DC voltage, which is named TET voltage for further description. In order to charge the battery pack with a maximum voltage of 14.4 V, the TET minimum voltage is determined to be 15 V. Depending on the displacement of the external and implanted coil and the load, the voltage varies between 15 and 50 V.

TET voltage and battery voltage were connected in parallel. The power switch ensures current flow in the direction of the driver electronics by efficient, actively switching diodes. This prevents a short circuit to the battery voltage in the rectifier and vice versa. Whenever the external TET system is connected, its DC output voltage will exceed the battery voltage and therefore supply the driver electronics. The permanent supply voltage is labeled control unit voltage. TET and battery voltage are constantly compared to each other to detect which input currently supplies the system.

To further improve the efficiency, no voltage converter is used to generate a constant control unit voltage. The power electronics of the motor driver, the battery, and the voltage level shifters, which create the operation voltages for sensors and microcontroller units, were configured to work with the input voltage range and regulate the output independent of the changing input. The resulting circuit is shown in area 1 of Figure 3.

Figure 4 illustrates how the TET voltage is connected to the charger of the battery pack. In order to assure supply of the control unit whenever the TET system is unable to deliver power, the battery will need to be charged as soon as the external TET System is connected. Constant-Current–Constant-Voltage charging is the optimum charging strategy for LiFePo_4_ batteries. The charging process is controlled by the microcontroller. Until the battery pack voltage reaches 14.4 V the entire battery pack is charged with constant current. After the battery, voltage reaches 14.4 V, the voltage is kept constant and the charging current slowly decreases. The battery is considered full when the charging current drops below 50 mA and the process is terminated by the software of the microcontroller. Over the charging period, the battery voltage increases continuously which roughly indicates the state of charge.

The sum of the cell voltages is not sufficient to ensure a safe charging process, since single cell voltages could exceed their end-of-charge voltage which could damage or even destroy the cell. Consequently, the voltages of all cells in the pack are balanced while charging to prevent overcharging of single battery cells and termination of the charging process when only one cell is fully charged. Through balancing, the maximum energy is saved in the battery pack. The cells are balanced passively by converting the excess charge to waste heat. The resulting charging and balancing circuit is presented in area 2 of Figure 3.

#### 3.1.2. Driver Electronics

**Figure 5:**
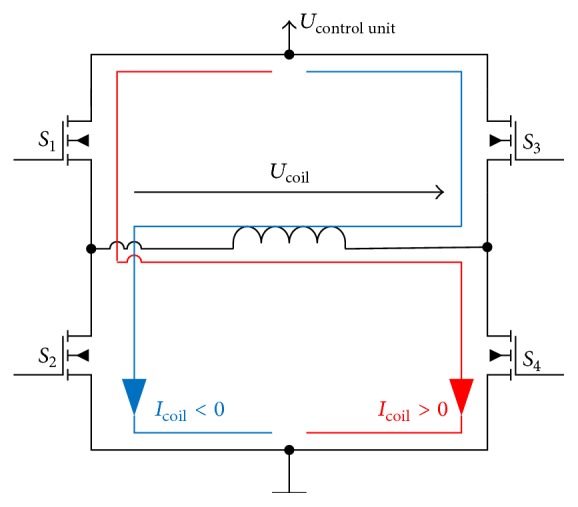
Schema of a full bridge circuit.

The moving part of the motor contains four coils. The current of each coil varies over time in sign and magnitude independent of the other coil currents. As such, each motor coil is driven by a separate electronic circuit, which is shown in Figure 5. The displayed full bridge allows the generation of positive or negative currents out of positive dc supply voltage.

Each of the full bridges for each coil is composed of four switches. If switches S1 and S4 are closed, positive current flows through the motor coil. On the contrary if switches S2 and S3 are closed, current flows in the opposite direction through the coil. Since the current in coils only changes slowly compared to the voltage across the coil, the desired magnitude of current can be created by fast chopping of the voltage across the coil. The time proportion in which the voltage is on, compared to the time period of a full cycle, determines the magnitude of the resulting current and should result in a linear relationship between “on” time and current.

#### 3.1.3. Microcontroller

The microcontroller provides input signals to the driver electronics and the battery charger. It also collects additional information from the other units like the control unit and battery voltage as well as the driver electronics total current, battery, and motor coil currents. It is important to gather the information in a single microcontroller to guarantee that the system is never running low on energy and detect fault conditions in the electronics before they become an issue for the patient.

**Figure 6:**
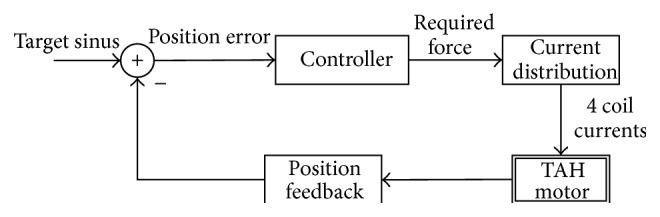
Position control schema.

Table 1 illustrates that the microcontroller is responsible for autonomous pumping. The motor in the pump unit is supposed to follow a sine wave with amplitude up to 9 mm to alternatingly empty the left and right pump chamber. In Figure 6, the algorithm controlling the motor's position is described: a position sensor in the TAH monitors the actual position of the pusher plates. This monitoring is critical, as deviations between the target and the actual position of the pusher plates may occur, due to the changing blood pressure. The position error is used to adapt the required force and maintain a smooth sinus motion. The force depends on the in- and outlet pressures of the ventricles as well as the pump speed. Since Lorenz force is directly proportional to the current flow through the motor coils and the fraction of the magnetic flux available in the coil's position, the current is distributed according to the strength of the magnetic field. This way no current is wasted into heat, in positions where no magnetic flux is available to create a force.

### 3.2. Validation of the Prototype

As described in Section 2, the pump unit was connected into the mock circulation loop and operated by the developed control unit for testing.

#### 3.2.1. Input Power

**Figure 7:**
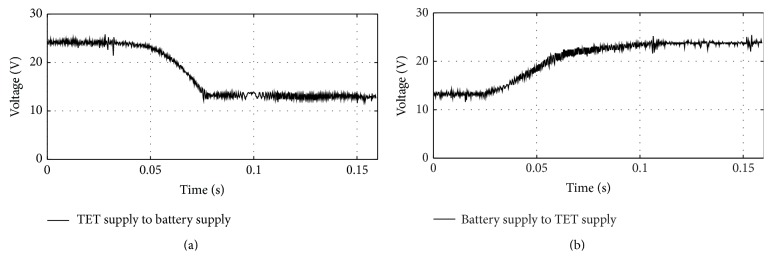
(a) Switching from TET supply to battery supply and (b) switching from battery supply to TET supply.

In a first trail, the uninterruptedly switching from TET to battery and back to TET was tested by continuous monitoring of the control unit voltage. The distance between the external and the implantable coil was set to 16 mm, which resulted in a 24 V TET voltage. The battery voltage was measured at 13 V. The control unit voltage at the transition from one input to another is shown in Figure 7.

When switching the TET system off, the control unit voltage dropped to the battery voltage within 30 ms. If the external coil is removed, the transition time highly depends on how fast the coil is moved. On battery support, no setback in performance was noticed and all pressures were kept at stable level. When the TET system was reconnected, the control unit voltage was constant at TET voltage level after about 50 ms. In summary, a smooth and fast transition between TET and battery voltage could be verified in both directions.

In another experiment, a battery pack was charged without balancing and the resulting cell voltages are detailed in the first column of Table 2. The maximum difference between two cells was found at 43 mV. In contrast, when integrating a balancer, voltages between any of the different battery packs were nearly identical. The absolute voltages in balanced state were smaller than the unbalanced values since no further charging took place during the balancing process. Conclusively, the implemented balancer assures that all cells of the battery pack are charged equally and therefore guarantees the maximum energy storage in the battery pack.

#### 3.2.2. Driver Electronics

**Figure 8:**
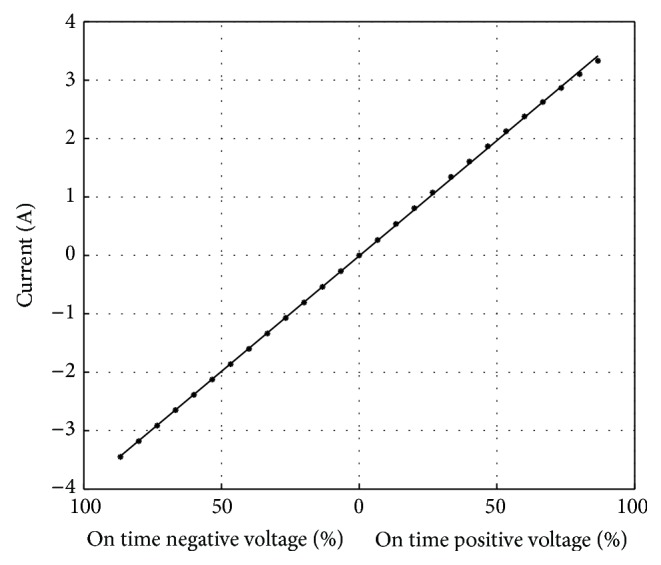
Current over “on” time of the voltage.

The suitability of the full bridge circuits to regulate the coil currents was tested in another study. In general, the “on” time of a chopped voltage across a motor coil determines the magnitude of the resulting current. This linear relationship was evaluated as displayed in Figure 8. For positive and negative voltage, 15 different percentages of on time were applied to the first coil of the motor and the resulting current through the coil was measured. The straight line represents a linear interpolation of the individual current measurements.

In short, all measured currents match the interpolation, which proves the linear relationship between “on” time of the chopped voltage and current in the motor coil. Consequently, the full bridges circuit is suitable to gradually control the coil current and ultimately control the motor movement.

#### 3.2.3. Microcontroller

**Figure 9:**
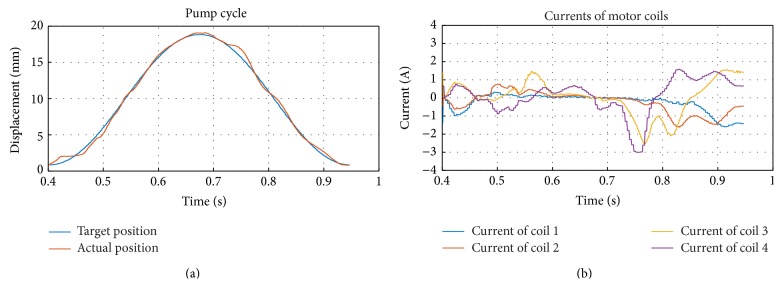
(a) Actual and target position among one pumping cycle and (b) current distribution in the four motor coils.

The position control was tested by comparing the target pusher plate position with the positions measured by the microcontroller (Figure 9). The first half of the pump cycle corresponds to the right systole and left diastole, while the second half corresponds to the left systole and right diastole. The actual position follows the target position with an error of up to 7.5%. Although the error is visible in the middle of the curve, the motor reaches the peak values at the end of the left and right systole and thereby guarantees the complete ejection of the ventricle volume into the arteries.

Figure 9 shows the four coil currents over one pumping cycle. As an illustrative case, coil 4 is observed during the left systole from 0.7 till 1 s. It starts with a high negative current at the beginning of the left systole. As the actuator with the attached coils moves through the magnetic field, the magnetic flux through coil 4 decreases, until it switches polarity at 0.71 s and continues to decrease. To maintain a force in the same direction, the current in coil 4 changes the polarity according to the change in the magnetic flux. It ends the left systole with a high positive current. Due to the higher pressure levels the currents during the left systole are generally higher than the currents during right systole. In summary, Figure 9 documents how the microcontroller successfully regulates the currents through all four coils depending on the magnetic field.

### 3.3. Overall Results of the Control Unit

**Figure 10:**
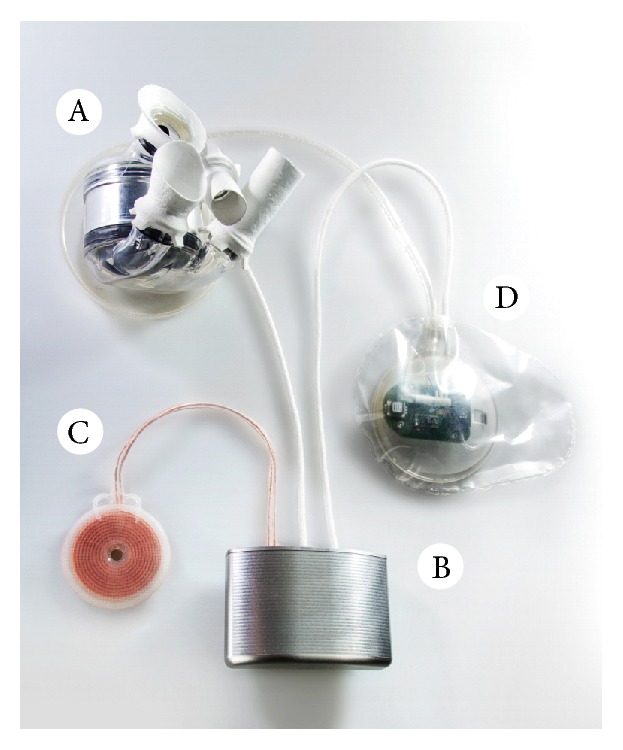
TAH implantable components: A-pump unit, B-first prototype of control unit, C-implanted TET coil, D-compliance chamber [5].

The implemented control unit is shown in Figure 10. Its outer dimensions are 125 mm length, 86 mm depths, and height between 25 and 37.5 mm due to the convex shape. The volume measures 360 cm^3^ and is comparable with the volume of earlier developments. The front panel holds three microjacks for connecting the implantable coil, the compliance chamber, and the pump unit. The jacks are sealed up to the front panel, which can be sealed to the housing itself.

**Figure 11:**
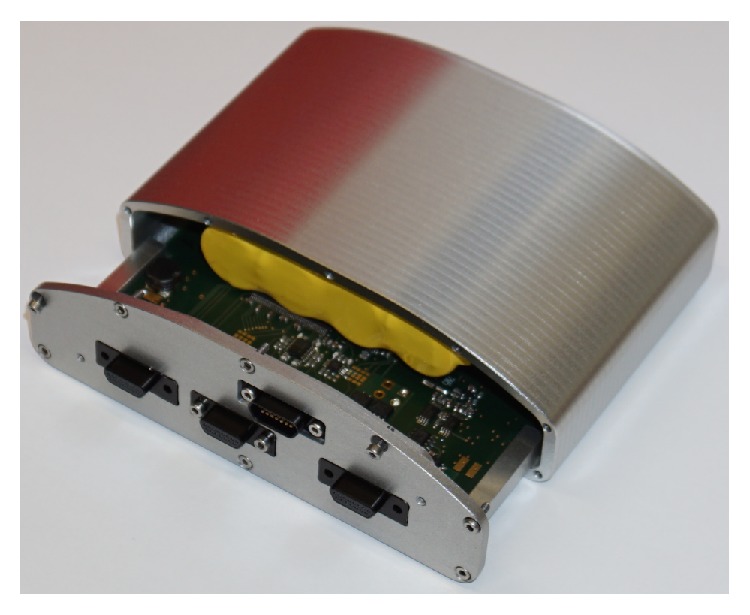
Front panel removed, view into the control unit.

In Figure 11, the inside of an opened control unit is displayed. The battery pack is fixed to the curved side of the case while the straight circuit board is mounted on the flat side of the case. In general, the size of the circuit board was adjusted to the length and width of the battery pack. However, some electrical components were too high to be mounted under the battery pack. Thus, the circuit board was extended in length to place higher electrical components aside from the battery pack. This size and shape of the control unit were validated positively in a cadaver study in a 75 kg male patient. Prototypes of the internal TET coil, the compliance chamber, and the control unit were positioned according to Figure 2. In comparison to a smaller dummy control unit with an additional battery pack in a second case, the one case prototype's fitting was satisfying, but allowed an easier implantation which is less traumatizing for the patient.

**Figure 12:**
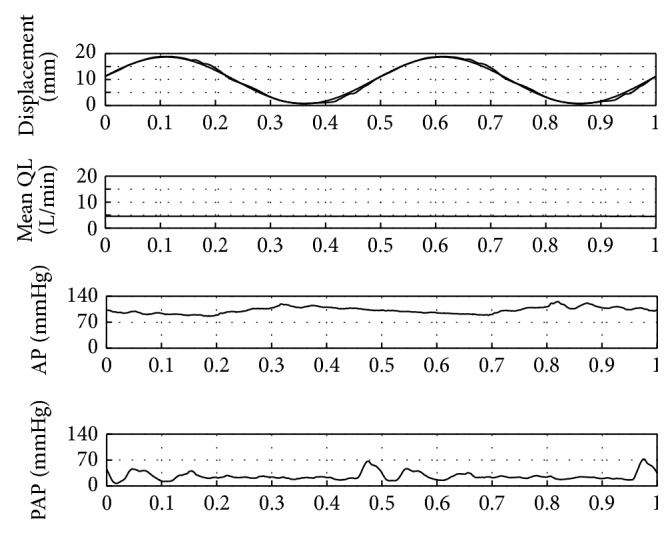
Hydraulic performance of the TAH.

Finally, hydrodynamic performance and efficiency of the complete TAH was investigated. The pressure in the compliance chamber was set up to a level which results in full fill, full ejection of the left and partial fill, and full ejection of the right pump chamber. This assures left right balance of the TAH flow and best hydraulic efficiency.  Figure 12 shows the flow and pressure curves captured by the DAQ for a beat rate of 120 bpm. The mean aortic flow was 5 L/min, which is comparable to blood flow of a healthy human. Mean aortic pressure and mean pulmonary artery pressure were 100 mmHg and 25 mmHg, respectively. The required mean power was 20 W.

Efficiencies for the various components were measured to evaluate how the losses are spread among the system. The efficiency of the control unit was calculated by the sum of the power dissipated in the four motor coils divided by the input electrical power drawn from the battery; the result was 83.5 % for the operating point. The efficiency of the hydraulic output power divided by the power drawn from the battery amounted to 22.6%. The hydraulic power was calculated by means of the mean flow rate and pressure levels. The average dc to dc efficiency of the TET system was evaluated and was determined to 62%. The peak efficiency at 45 W was 74%. Thereby the average efficiency of the control unit when supplied by the TET system would be 14%.

Further beat rates were investigated and pump and control unit performed in the full operating range up to 160 bpm and provided a maximum mean flow over 7.5 L/min.

## 4. Conclusion

A control unit which satisfies the requirements for a fully implantable TAH was designed and validated. It allows a safe and efficient operation of the designated pump unit of the ReinHeart TAH. The control unit successfully operated the pump unit without interruption during switchover between battery and TET in in vitro tests. The microcontroller software achieved autonomous pumping by controlling the motors motion on a sinusoidal trajectory. The utilized state of the art microcontroller technology enables modifications of the target trajectory to improve the interaction with the patient's physiology. It collects data from all modules which offers the implementation of safety queries and complex control algorithms. This variety of software adjustments was not implemented in earlier devices. Although a prototype for animal trails was accomplished, the efficiency and geometric dimensions were kept in a range acceptable for human implantation as experience with earlier devices indicate. The electronics for the TET system, the battery, and the motor control were inserted in one case. Compared to AbioCor TAH which used separate casing for the battery, the implantation expense was reduced. A cadaver study in a 75 kg male patient proved good fitting of the control unit prototype.

Some parts of the control unit already performed in animal trials and durability tests. Since all implantable components are designed for a lifetime of five years to bridge a reasonable timeframe, further in vitro and in vivo studies, especially long term trials, are necessary to confirm the performance of the entire control unit.

After long term validation of the TAH control unit additional improvements should be aimed for. The efficiency of the system can be improved by improving the control loop. This issue will be addressed in near future.

A wireless communication would allow forwarding the data collected by the microcontroller to an external user interface. Thereby the physician in charge could control the TAH operation. An ultimate goal would be physiological control of the pump rate according to the patient's blood pressures.

In a next integration step, the size of the control unit will be further reduced by applying state of the art micro technologies for circuit boards. Future battery technology will increase the support time of the internal batteries.

The implemented prototype was especially designed for the specific motor of the ReinHeart TAH. The same setup could be used to control active magnetic bearings of fully implantable blood pumps. Alternatively, the driver electronics of the setup could be easily adapted to drive a three phase rotatory device. In, the input power organization described in this paper is suitable for all fully implantable pumps.

## Figures and Tables

**Table 1:** Influence of the different requirements on each part of the control unit.

	Input power	Driver electronics	Microcontroller

Wireless safety	Add rectifier Add battery	Adapted to wide voltage input range	Monitor TET voltage Monitor battery voltage

Autonomous	Automatic switching, recharging as soon as TET is connected	Provide coil currents	Control of the motor trajectory by control of the motor currents

Efficiency	Utilization of small number of efficient components with low heat production		

Geometry	Utilization of small packages and low number of electrical components		

**Table 2:** Differences in cell voltages for balanced and unbalanced charging.

	Unbalanced voltage [V]	Balanced voltage [V]

Battery cell 1	3.584	3.441
Battery cell 2	3.588	3.442
Battery cell 3	3.595	3.443
Battery cell 4	3.552	3.442

## References

[1] Gerosa G., Scuri S., Iop L. (2014). Present and future perspectives on total artificial hearts. *Annals of Cardiothoracic Surgery*.

[2] Demondion P., Fournel L., Niculescu M., Pavie A., Leprince P. (2013). The challenge of home discharge with a total artificial heart: the La Pitié Salpêtrière experience. *European Journal of Cardio-Thoracic Surgery*.

[3] Dowling R. D., Gray L. A., Etoch S. W. (2003). The AbioCor implantable replacement heart. *The Annals of Thoracic Surgery*.

[4] Deutsch S., Tarbell J. M., Manning K. B., Rosenberg G., Fontaine A. A. (2006). Experimental fluid mechanics of pulsatile artificial blood pumps. *Annual Review of Fluid Mechanics*.

[5] Pelletier B., Spiliopoulos S., Finocchiaro T. (2015). System overview of the fully implantable destination therapy—ReinHeart-total artificial heart. *European Journal of Cardio-Thoracic Surgery*.

[6] Finocchiaro T., Butschen T., Kwant P. (2008). New linear motor concepts for artificial hearts. *IEEE Transactions on Magnetics*.

[7] Oman H. (2002). Artificial hearts, batteries, and electric vehicles. *IEEE Aerospace and Electronic Systems Magazine*.

[8] Kustosz R., Brandt J., Szczurek Z., Kaczmarczyk Z., Stepien M., Grzesik B. Charging and discharging backup battery for artificial heart.

[9] Tatsumi E., Taenaka Y., Homma A. (2003). The National Cardiovascular Center electrohydraulic total artificial heart and ventricular assist device systems: current status of development. *ASAIO Journal*.

[10] Takatani S., Sakamoto T., Ohuchi K., Nakamura M., Mizuno T., Arai H. (2002). One piece ultracompact totally implantable electromechanical total artificial heart for permanent use. *ASAIO Journal*.

[11] Fritschi A. J., Laumen M., Spiliopoulos S. (2013). Image based evaluation of mediastinal constraints for the development of a pulsatile total artificial heart. *BioMedical Engineering Online*.

[12] Homma A., Taenaka Y., Tatsumi E. Current status of the National Cardiovascular Center totally implantable artificial heart system.

[13] Cuenca-Navalon E., Finocchiaro T., Laumen M., Fritschi A., Schmitz-Rode T., Steinseifer U. (2014). Design and evaluation of a hybrid mock circulatory loop for total artificial heart testing. *The International Journal of Artificial Organs*.

[14] Župa T., Líška O. Charging module for newest types of rechargeable batteries LiFePO_4_.

